# Supplementary material for: Local and systemic effect of transfection-reagent formulated DNA vectors on equine melanoma
Source: BMC Vet Res. 2015 May 14;11:107. doi: 10.1186/s12917-015-0414-9 (PMC4429833; doi:10.1186/s12917-015-0414-9)
Supplement: Additional file 1: — Contains supplementary text file 1. [file 12917_2015_414_MOESM1_ESM.docx]

**Appendix A**

1. Ligation of the signal peptide gene of the IL1beta receptor antagonist protein (ILRAP) to the IL18 gene

In order to produce a more effective, secreted form of IL18, the IL18 sequence was modified by ligating the oligonucleotides ILRAP fw1 (CAT GGA AAT CTG CAG AGG CCT CCG CAG TCA CCT AAT CAC), -fw2 (TCT CCT CCT CTT CCT GTT CCA TTC AGA GAC GAT CTG CCT GC), -rv1(TTA GGT GAC TGC GGA GGC CTC TGC AGA TTT CCA TGG TAC) and rv 2 (GCA GAT CGT CTC TGA ATG GAA CAG GAA GAG GAG GAG AGT GA) (Microsynth, Balgach, Switzerland) coding for the IL1beta receptor antagonist protein (ILRAP) signal sequence to the original eqIL18 gene. Dephosphorylated oligonucleotides were diluted to 100 μM using tri-distilled water. Phosphorylation was performed by incubation of 23 μl oligonucleotides ILRAP rv-1 and fw2 with 3 μl polynucleotidkinase (PNK) A buffer (MBI Fermentas, Vilnius, Lithuania), 1 μl PNK and 3 μl ATP at 37 °C for one hour. Enzyme was inactivated subsequently at 75 °C for 20 minutes. By adding 23 μl of dephosporylated oligonucleotides to the phosphorylated oligonucleotides (ILRAP rv1 with ILRAP fw1; ILRAP fw2 with ILRAP rv2) annealing was performed by incubation for three minutes at 75 °C. Intended for ligation, the annealed oligonucleotides were incubated for one hour with 10 μl ligase buffer, 10 μl ATP, and 2 μl T4 ligase at room temperature.

1. Detection of antibodies

To measure specific antibodies against hgp100 and htyr in the sera of the patients a cell based, flow cytometric antibody assay was performed. Therefore, sera from the patients were obtained before each injection and on day 120. The sera were incubated with hgp100 and tyr transfected HEK293 cells. Evaluation was performed after staining bounded antibodies with a secondary antibody by flow cytometry.

In detail, blood was collected from the jugular vein of all patients on days 1, 22, 78 and 120. The blood samples were allowed to clot for 30 to 40 minutes, centrifuged (3500 *g*, 10 min), and the serum frozen at -80 °C. Prior analysis, the serum was thawed to room temperature and immediately tested thereafter. To provide the antigen, HEK 293 cells, grown in HEK-culture medium (Minimal Essential Medium Eagle (MEM), supplemented with 10% heat-inactivated fetal calf serum (FCS), 1% non-essential amino acids, 1% pyruvate, penicillin (100 IU/ml), streptomycin (100 μg/ml), all PAA Laboratories GmbH, Pasching, Austria) and maintained at 37°C with 5% CO_2_, were transfected with pMCV1.4 htyr resp. pMCV1.4 hgp100 plasmid using Lipofectamine LTX (Invitrogen, Darmstadt, Germany) according to the manufacturer’s instructions. After 24 hours the HEK293 cells were dissociated using 1ml Accutase^®^ (PAA Laboratories GmbH, Pasching, Austria) per 75 cm^2^, counted and resuspended in FACS-buffer to a final concentration of 10^6^ cells/ml. In each well of a round-bottom 96-well-plate (Gechno Plastic Products, TPP^®^, Trasadingen, Switzerland) 2x10^5^ HEK293 (untransfected or either transfected with hgp100 or htyr) were plated. Cells were fixed with 200μl of 4% paraformaldehyde (Carl Roth GmbH&Co.KG, Karlsruhe, Germany) per well for 30 minutes at room temperature (RT) and washed three times with PBS. Sera were diluted in saponin-buffer to 1:1600. Fifty μl were added to the cells and incubated for 30 minutes at RT. Each serum was separately tested for antibodies against hgp100 and htyr using the hgp100 and htyr expressing HEK293 cells. After washing the cells 3 times with saponin-buffer the secondary antibody goat anti horse fluorescein isothiocyanate (FITC) (Jackson ImmunoResearch Laboratories, West Grove, USA) was added, followed by incubation for 30 min at RT and three washing steps with saponin-buffer. Monoclonal antibodies specific for hgp100 resp. htyr (Monoclonal MouseAnti-Human Melanosome Clone HMB45, Monoclonal Mouse Anti-Human Tyrosinase Clone T311, both Dako North America Inc., Carpinteria, CA, USA), diluted 1:200 in saponin-buffer served as positive controls. Non-transfected cells incubated with the tested sera, were used as negative controls.

Stained cells were washed twice with saponin-buffer and resuspended in 200 μl of Sheath-buffer. In flow cytometry 10.000 events were counted (FACScan^®^, CellQuest™ Pro 4.0.2, Beckton Dickinson, Heidelberg, Germany). The fluorescence intensity of stained cells was analyzed using FcsExpress software (De Novo Software Los Angeles, CA, USA).
