# Supplementary figures and images for: Local and systemic effect of transfection-reagent formulated DNA vectors on equine melanoma
Source: BMC Vet Res. 2015 May 14;11:107. doi: 10.1186/s12917-015-0414-9 (PMC4429833; doi:10.1186/s12917-015-0414-9)

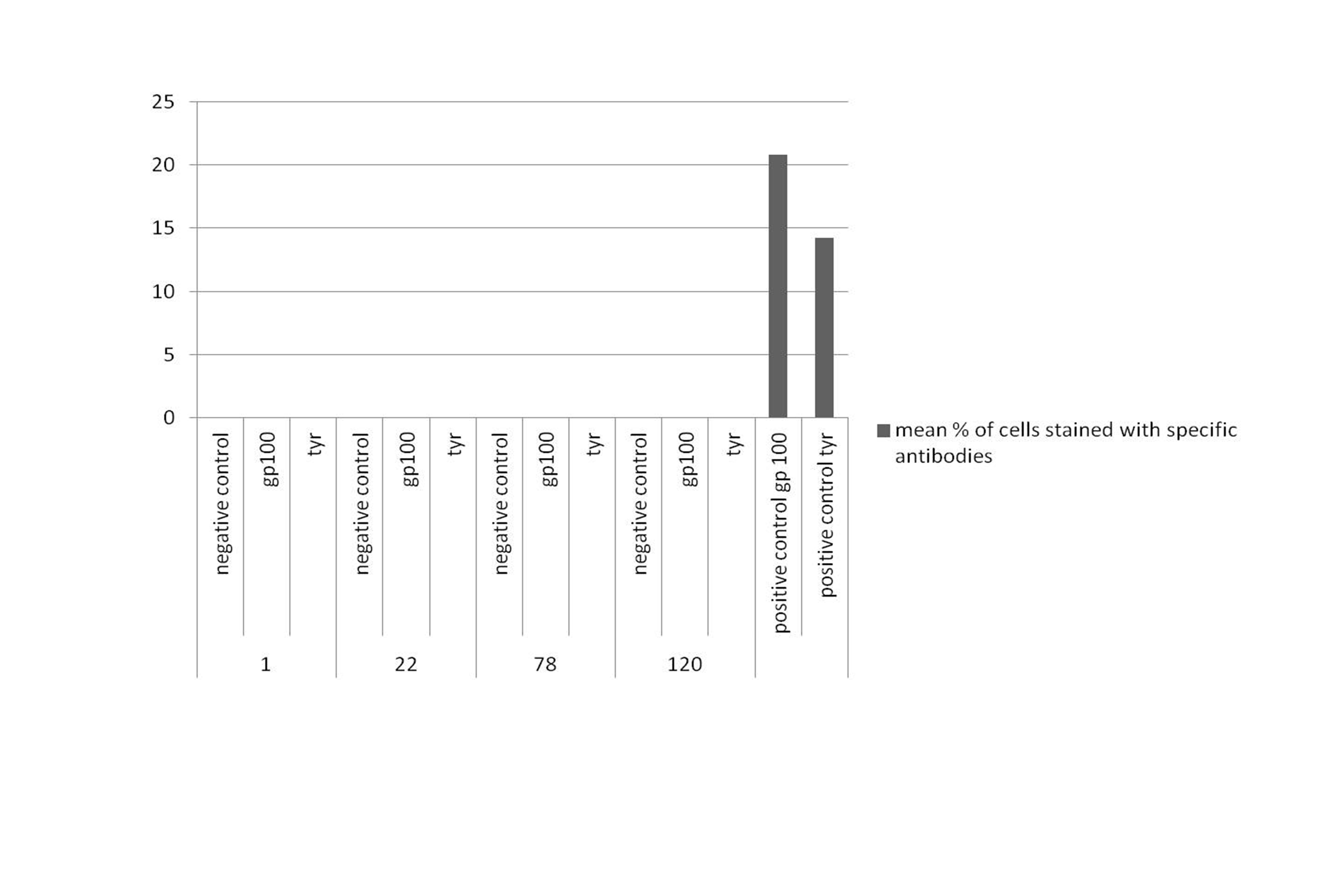

Supplement: Additional file 2: Figure S1. — No specific antibodies were detected in serum samples from vaccinated horses on days 1, 22, 78 and 120. [file 12917_2015_414_MOESM2_ESM.jpeg]
